# Supplementary material for: Ocelli: an open-source tool for the analysis and visualization of developmental multimodal single-cell data
Source: NAR Genom Bioinform. 2025 Apr 10;7(2):lqaf040. doi: 10.1093/nargab/lqaf040 (PMC12086682; doi:10.1093/nargab/lqaf040)
Supplement: lqaf040_Supplemental_File [file lqaf040_supplemental_file.pdf]

Supplementary information for: "Ocelli: an open-source tool for the analysis and visualization of developmental multimodal single-cell data"

Preprocessing and analysis details of single-cell datasets

Hair follicle differentiation (SHARE-seq)

The hair follicle dataset is a subset of a SHARE-seq dataset [1] for female mouse dorsal skin and contains 7,160 cells. We generated the RNA count matrix from BAM files using Velocyto v0.17.17 [2], producing the gene expression matrix with unspliced and spliced layers. We kept transit-amplifying cells (labeled *TAC-1*, *TAC-2*), inner root sheath cells (labeled *IRS*), and hair shaft cells (labeled *Medulla* and *Hair Shaft-cuticle.cortex*). When filtering the expression matrix, we performed the following steps: (i) removed Malat1, Gm42418, and AY036118 genes associated with RNA contamination; (ii) removed cell doublets using Scrublet v0.2.3 [3] with a predefined estimated collision rate [1]; (iii) removed cells with less than 50 expressed genes; (iv) removed genes with less than 50 counts; (v) removed genes highly correlated with the gene signature of cell proliferation [4]. The preprocessing steps have been carried out with Scanpy v1.9.1 [5]. In step (v), we computed Pearson correlations between the log-normalized expression of genes and the mean z-scores of the proliferation gene signature. Genes with a Pearson correlation larger than 0.18 were discarded. The resulting RNA count matrix had 7,160 cells and 6,731 genes. Then, we computed 50 LDA topics (scikit-learn v1.0.2 [6]) of the count matrix and scVelo's v0.2.4 [7] stochastic RNA velocity transitions on 1,000 log-normalized highly variable genes.

We generated the ATAC-seq count matrix using Signac v1.10.0 [8] by mapping chromatin accessibility fragments to gene regions, including the 2kb upstream region. Next, we filtered out genes with less than 50 counts and genes correlated with the proliferation gene signature (the same genes as in RNA-seq). The resulting ATAC count matrix had 7,160 cells and 17,495 genes. Then, we computed 50 LDA topics of the count matrix.

During the multimodal analysis with Ocelli, we (i) reduced both modalities to 50 LDA topics, (ii) computed a 20-dimensional MDM embedding using 20 nearest neighbors, (iii) represented the MDM latent space in 3 dimensions using a 3-edge RNA velocity-based graph with ForceAtlas2, and (iv) projected the 3-dimensional embedding onto 2 dimensions. The gene signatures for annotation of terminally differentiated cells were taken from SMART-seq2 Ana-VI hair follicle dataset [9]. Cuticle cell gene signature scores from IRS and HS [9] overlapped with Huxley's and Cortex gene signature scores, respectively. The Cuticle cells in high-throughput scRNA-seq methods were indistinguishable from Cortex or IRs cells [1, 10] and were annotated jointly with them. For the sake of simplicity, we used only Huxley's and Cortex annotation labels for cell identification.

Native hematopoietic differentiation (ASAP-seq)

The human bone marrow ASAP-seq dataset [11] contains 10,927 cells. We used matrices preprocessed by the dataset authors, namely, the protein count matrix with 238 protein tags and the ATAC-seq gene activity matrix with 3,000 genes (chromatin accessibility fragments were mapped to gene regions, including the 2kb upstream region, using Signac [8]).

During the multimodal analysis with Ocelli, we (i) reduced both modalities to 15 LDA topics, (ii) computed a 10-dimensional MDM embedding using 70 nearest neighbors, (iii) represented the MDM latent space in 2 dimensions using a 20-nearest neighbor graph with ForceAtlas2. When analyzing the differentiation of hematopoietic stem cells (HSCs), we created a vector with 1s assigned to HSCs and 0s to the remaining cells. After running multimodal diffusion on the HSCs vector for $t = 20$ steps, we selected 1,464 cells. Then, we (i) reduced both modalities to 15 LDA topics, (ii) computed a 7-dimensional MDM embedding using 50 nearest neighbors, and (iii) reduced the MDM latent space in 2 dimensions using a 50-nearest neighbor graph with ForceAtlas2.

Native hematopoietic differentiation (NTT-seq)

The human bone marrow NTT-seq dataset [12] contains 5,236 cells. We generated count matrices of histone modification profiles H3K27ac (71,253 peaks) and H3K27me3 (43,170 peaks) using Signac [8].

During the multimodal analysis with Ocelli, we (i) reduced both modalities to 20 LDA topics, (ii) computed a 10-dimensional MDM embedding using 100 nearest neighbors, and (iii) represented the MDM latent space in 2 dimensions using a 20-nearest neighbor graph with ForceAtlas2.

Native hematopoietic differentiation (SHARE-seq)

The human bone marrow SHARE-seq dataset [13] contains 842,440 cells. We filtered the RNA count matrix by removing cells with less than 100 expressed genes and genes expressed in less than 100 cells, resulting in 33,721 genes. We selected cells in the ATAC count matrix to match filtered RNA cells and left all 189,259 peaks.

During the multimodal analysis with Ocelli, we (i) reduced both modalities to 50 LDA topics, (ii) computed a 50-dimensional MDM embedding using 100 nearest neighbors, and (iii) represented the MDM latent space in 2 dimensions using a 30-nearest neighbor graph with ForceAtlas2.

Peripheral blood mononuclear cells (DOGMA-seq)

The peripheral blood mononuclear cells dataset is a control subset of a DOGMA-seq dataset [11] under low-loss lysis (LLL) condition and contains 7,516 cells. We filtered cells with less than 1,000 ATAC counts, less than 1,000 RNA counts, less than 500 RNA genes expressed, and less than 25,000 surface protein counts. The preprocessed count matrices have 84,846 peaks (ATAC), 36,601 genes (RNA), and 210 surface proteins.

During the multimodal analysis with Ocelli, we (i) reduced both modalities to 20 LDA topics, (ii) computed a 20-dimensional MDM embedding using 100 nearest neighbors, and (iii) represented the MDM latent space in 2 dimensions using a 20-nearest neighbor graph with ForceAtlas2.

Pancreatic endocrinogenesis (scRNA-seq)

The pancreatic endocrinogenesis RNA-seq dataset [14] contains 3,696 cells sampled from embryonic day 15.5. We used the published [7] gene expression matrix with unspliced and spliced layers. We filtered out cells expressed in less than 20 genes and removed genes expressed in less than 20 cells in both unspliced and spliced layers. The resulting count matrix has 5,465 genes. We computed RNA velocity with scVelo [7] on 1,000 log-normalized highly variable genes.

During the analysis with Ocelli, we (i) computed 20 LDA topics on the count matrix, (ii) generated topic-based modalities, (iii) computed a 25-dimensional MDM embedding using 20 nearest neighbors, and (iv) represented the MDM latent space in 2 dimensions using a 20-edge RNA velocity-based graph with ForceAtlas2.

Induced pluripotent stem cell reprogramming (scRNA-seq)

The induced pluripotent stem cell reprogramming scRNA-seq dataset [4] contains 68,703 cells. We generated the count matrix from BAM files using Velocyto [2]. When filtering the count matrix, we carried out the following steps: (i) kept cells from day 0 to 8 and cells in serum condition (from day 8.25 to 18); (ii) removed doublets using Scrublet [3] with a doublet score threshold set to 0.3; (iii) maintained cells from days 8-18; (iv) removed cells with less than 2,000 UMIs; (v) removed genes expressed in less than 50 cells; (vi) downsampled cells to 15,000 UMIs. Steps (iv)-(vi) followed the preprocessing instructions from the dataset authors. The resulting count matrix has 16,817 genes.

During the analysis with Ocelli, we (i) computed 20 LDA topics on the count matrix, (ii) generated topic-based modalities, (iii) computed a 20-dimensional MDM embedding using 20 nearest neighbors, (iv) represented the MDM latent space in 3 dimensions using a 10-edge WOT transitions-based graph (with cell timestamps) with ForceAtlas2, and (v) projected the 3-dimensional embedding onto 2 dimensions.

Data preprocessing for benchmarking

This section describes generating embeddings for cohesiveness, RNA velocity vector field alignment, and RNA velocity-based terminal state separation benchmarks. We employed the following preprocessing procedures, following the recommendations of method authors. (*Cobolt*) We trained 20-dimensional latent spaces for 200 epochs on (i) 1,000 highly variable RNA-seq genes and ATAC-seq peaks detected in more than 1 % of cells; (ii) all RNA-seq genes and ATAC-seq peaks detected in more than 1 % of cells. Additionally, we set the learning rate 0.001 and 0.0001. (*Matilda*) We trained 20-dimensional latent spaces for 200 epochs on (i) 1,000 highly variable RNA-seq genes and 1,000 highly variable ATAC-seq gene activities, (ii) all RNA-seq genes, and all ATAC-seq gene activities. Additionally, we set the learning rate 0.001 and 0.0001. (*MIRA*) We trained 20-dimensional latent spaces on (i) 1,000 highly variable RNA-seq genes and ATAC-seq peaks detected in more than 1 % of cells; (ii) all RNA-seq genes and ATAC-seq peaks detected in more than 1 % of cells. Additionally, we set the number of encoder layers to 3 and 4, and the number of nodes per layer to 64, 128, and 256. (*MOFA+*) We trained 20-dimensional latent spaces in slow (most accurate) convergence mode on (i) 1,000 highly variable RNA-seq genes and 1,000 highly variable ATAC-seq gene activities; (ii) all RNA-seq genes and all ATAC-seq gene activities. Each modality was scaled to a unit variance and models used Gaussian likelihoods. (*MOJITOO*) We trained latent spaces on (i) 50 PCs of log-normalized 1,000 highly variable RNA-seq genes and 50 PCs of log-normalized 1,000 highly variable ATAC-seq gene activities; (ii) 50 PCs of log-normalized all RNA-seq genes and 50 PCs of log-normalized all ATAC-seq gene activities. The dimensionality of latent spaces was computed automatically, resulting in (i) 40 and (ii) 41 dimensions. (*MultiVI*) We trained 20-dimensional latent spaces for 200 epochs on (i) 1,000 highly variable RNA-seq genes and ATAC-seq peaks detected in more than 1 % of cells; (ii) all RNA-seq genes and ATAC-seq peaks detected in more than 1 % of cells. Additionally, we set the modality weights parameter to *cell*, *equal*, and *universal*. (*Ocelli*) We trained 20-dimensional latent spaces on 50 LDA topics trained on all RNA-seq genes and 50 LDA topics trained on all ATAC-seq gene activities. Additionally, we performed nearest neighbor searches using both exact (ENNs) and approximate (ANNs) methods. Transition probabilities were computed using scVelo [7] (stochastic model trained 1,000 on highly variable genes) and MultiVelo [15] (stochastic and dynamical models trained on the same set of highly variable RNA genes overlapping with ATAC gene activities). (*scMM*) We trained 20-dimensional latent spaces for 200 epochs on (i) 1,000 highly variable RNA-seq genes and ATAC-seq peaks detected in more than 1 % of cells; (ii) all RNA-seq genes and ATAC-seq peaks detected in more than 1 % of cells. Additionally, we set the number of encoder layers to 1, 2, and 3, and the learning rate to 0.001 and 0.0001. (*WNN*) We trained multimodal graphs on (i) 50 PCs of log-normalized 1,000 highly variable RNA-seq genes and 50 PCs of log-normalized 1,000 highly variable ATAC-seq gene activities, (ii) 50 PCs of log-normalized all RNA-seq genes and 50 PCs of log-normalized all ATAC-seq gene activities. Additionally, we set the number of nearest neighbors to 20, 50, and 100. The resulting latent spaces and graphs were reduced to 2 and 3 dimensions with UMAP using 20 nearest neighbors and a minimum distance of 0.1. We additionally computed 2- and 3-dimensional ForceAtlas2 embeddings for Ocelli using a 3-edge RNA velocity-based graphs. We generated each embedding for 10 random seeds and present mean benchmark scores to negate any stochastic effects of dimension reduction algorithms on benchmark results. Unless otherwise stated, we present only scores for best-performing parameters for each method.

References

1. Sai Ma, Bing Zhang, Lindsay M LaFave, Andrew S Earl, Zachary Chiang, Yan Hu, Jiarui Ding, Alison Brack, Vinay K Kartha, Tristan Tay, et al. Chromatin potential identified by shared single-cell profiling of RNA and chromatin. *Cell*, 183(4):1103–1116, 2020.
2. Gioele La Manno, Ruslan Soldatov, Amit Zeisel, Emelie Braun, Hannah Hochgerner, Viktor Petukhov, Katja Lidschreiber, Maria E Kastriti, Peter Lönnerberg, Alessandro Furlan, et al. RNA velocity of single cells. *Nature*, 560(7719):494–498, 2018.
3. Samuel L Wolock, Romain Lopez, and Allon M Klein. Scrublet: computational identification of cell doublets in single-cell transcriptomic data. *Cell Syst.*, 8(4):281–291, 2019.
4. Geoffrey Schiebinger, Jian Shu, Marcin Tabaka, Brian Cleary, Vidya Subramanian, Aryeh Solomon, Joshua Gould, Siyan Liu, Stacie Lin, Peter Berube, et al. Optimal-transport analysis of single-cell gene expression identifies developmental trajectories in reprogramming. *Cell*, 176(4):928–943, 2019.
5. F Alexander Wolf, Philipp Angerer, and Fabian J Theis. SCANPY: large-scale single-cell gene expression data analysis. *Genome Biol.*, 19:1–5, 2018.
6. F. Pedregosa, G. Varoquaux, A. Gramfort, V. Michel, B. Thirion, O. Grisel, M. Blondel, P. Prettenhofer, R. Weiss, V. Dubourg, J. Vanderplas, A. Passos, D. Cournapeau, M. Brucher, M. Perrot, and E. Duchesnay. Scikit-learn: Machine learning in Python. *J. Mach. Learn. Res.*, 12:2825–2830, 2011.
7. Volker Bergen, Marius Lange, Stefan Peidli, F Alexander Wolf, and Fabian J Theis. Generalizing RNA velocity to transient cell states through dynamical modeling. *Nat. Biotechnol.*, 38(12):1408–1414, 2020.
8. Tim Stuart, Avi Srivastava, Shaista Madad, Caleb A Lareau, and Rahul Satija. Single-cell chromatin state analysis with Signac. *Nat. Methods*, 18(11):1333–1341, 2021.
9. Hanseul Yang, Rene C Adam, Yejing Ge, Zhong L Hua, and Elaine Fuchs. Epithelial-mesenchymal micro-niches govern stem cell lineage choices. *Cell*, 169(3):483–496, 2017.
10. Simon Joost, Karl Annusver, Tina Jacob, Xiaoyan Sun, Tim Dalessandri, Unnikrishnan Sivan, Inês Sequeira, Rickard Sandberg, and Maria Kasper. The molecular anatomy of mouse skin during hair growth and rest. *Cell Stem Cell*, 26(3):441–457, 2020.
11. Eleni P Mimitou, Caleb A Lareau, Kelvin Y Chen, Andre L Zorzetto-Fernandes, Yuhan Hao, Yusuke Takeshima, Wendy Luo, Tse-Shun Huang, Bertrand Z Yeung, Efthymia Papalexi, et al. Scalable, multimodal profiling of chromatin accessibility, gene expression and protein levels in single cells. *Nat. Biotechnol.*, 39(10):1246–1258, 2021.
12. Tim Stuart, Stephanie Hao, Bingjie Zhang, Levan Mekerishvili, Dan A Landau, Silas Maniatis, Rahul Satija, and Ivan Raimondi. Nanobody-tethered transposition enables multifactorial chromatin profiling at single-cell resolution. *Nat. Biotechnol.*, pages 1–7, 2022.
13. Yan Hu, Sai Ma, Vinay K Kartha, Fabiana M Duarte, Max Horlbeck, Ruochi Zhang, Rojesh Shrestha, Ajay Labade, Heidi Kletzien, Alia Meliki, et al. Single-cell multi-scale footprinting reveals the modular organization of dna regulatory elements. *bioRxiv*, 2023.
14. Aimée Bastidas-Ponce, Sophie Tritschler, Leander Dony, Katharina Scheibner, Marta Tarquis-Medina, Ciro Salinno, Silvia Schirge, Ingo Burtscher, Anika Böttcher, Fabian Theis, Heiko Lickert, and Mostafa Bakhti. Massive single-cell mRNA profiling reveals a detailed roadmap for pancreatic endocrinogenesis. *Development*, 146:dev.173849, 2019.
15. Chen Li, Maria C Virgilio, Kathleen L Collins, and Joshua D Welch. Multi-omic single-cell velocity models epigenome–transcriptome interactions and improves cell fate prediction. *Nat. Biotechnol.*, 41(3):387–398, 2023.
16. Tal Ashuach, Mariano I Gabitto, Rohan V Koodli, Giuseppe-Antonio Saldi, Michael I Jordan, and Nir Yosef. MultiVI: deep generative model for the integration of multimodal data. *Nat. Methods*, pages 1–10, 2023.

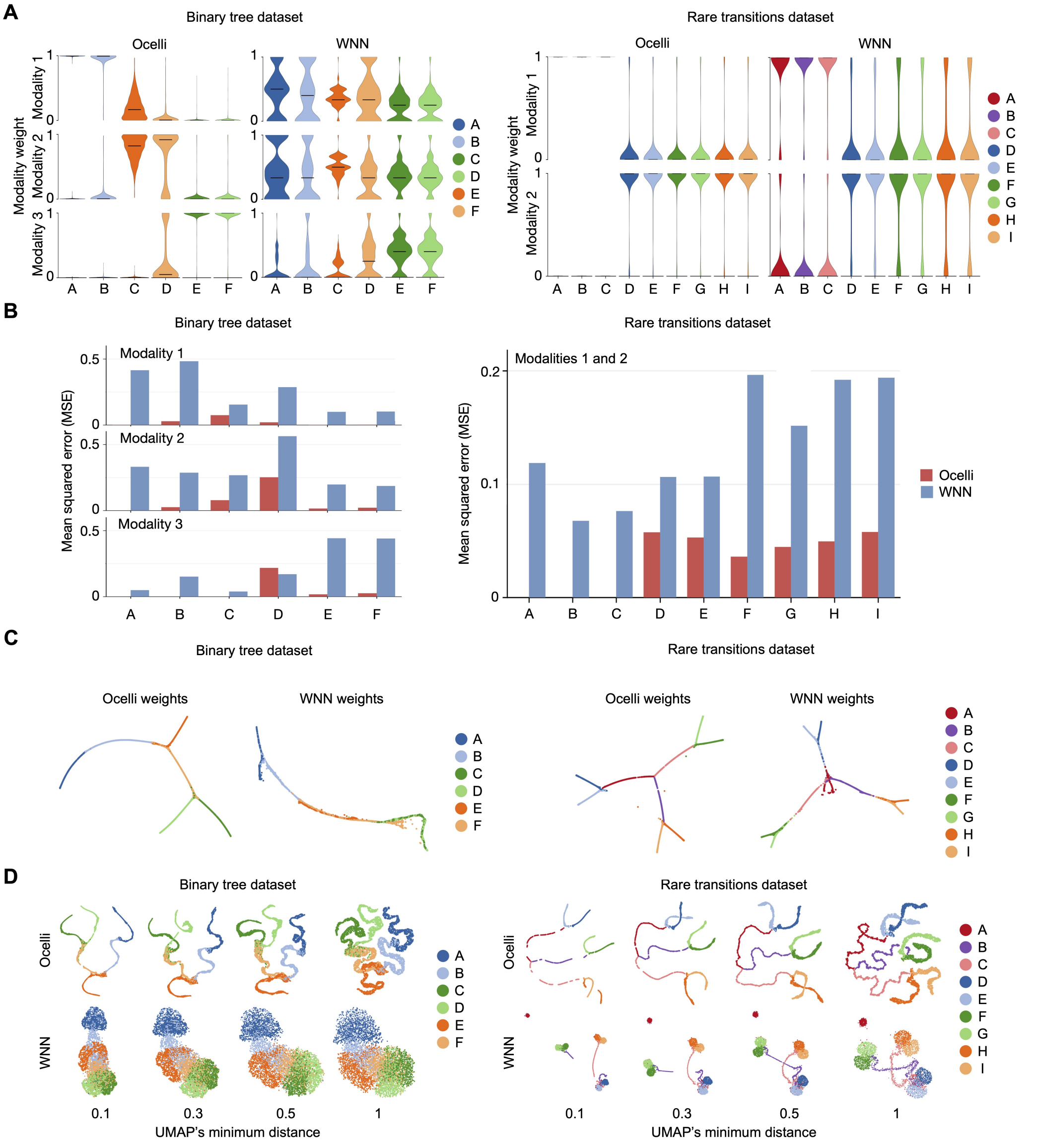

Figure S1. Reconstruction of developmental trajectories in simulated data with Ocelli (related to Figure 1). Additional exploration of the binary tree dataset (see Figure 1B) consisting of three modalities and six developmental lineages and the rare transitions dataset (see Figure 1C) consisting of two modalities and nine lineages. **(A)** Distributions of inferred multimodal weights computed using Ocelli and WNN. Violin plots show how informative modalities are for each cell lineage according to both methods. In the binary tree dataset, modalities 1, 2, and 3 are most informative about developmental lineages A-B, C-D, and E-F, respectively. In the rare transitions dataset, modalities 1 and 2 are most informative about developmental lineages A-C and D-I, respectively. **(B)** Comparison of target and inferred weights computed using Ocelli and WNN. Target modality weights equal 1 for lineages introduced in the modality and 0 for the remaining lineages. Mean squared error (MSE) measures the dissimilarity between inferred and target weights. A lower error indicates a better sensitivity of weights. **(C)** Ocelli embeddings visualized using ForceAtlas2 computed with modality weights from either Ocelli or WNN. **(D)** Ocelli, and WNN embeddings visualized using UMAP with increasing values of the minimum distance parameter.

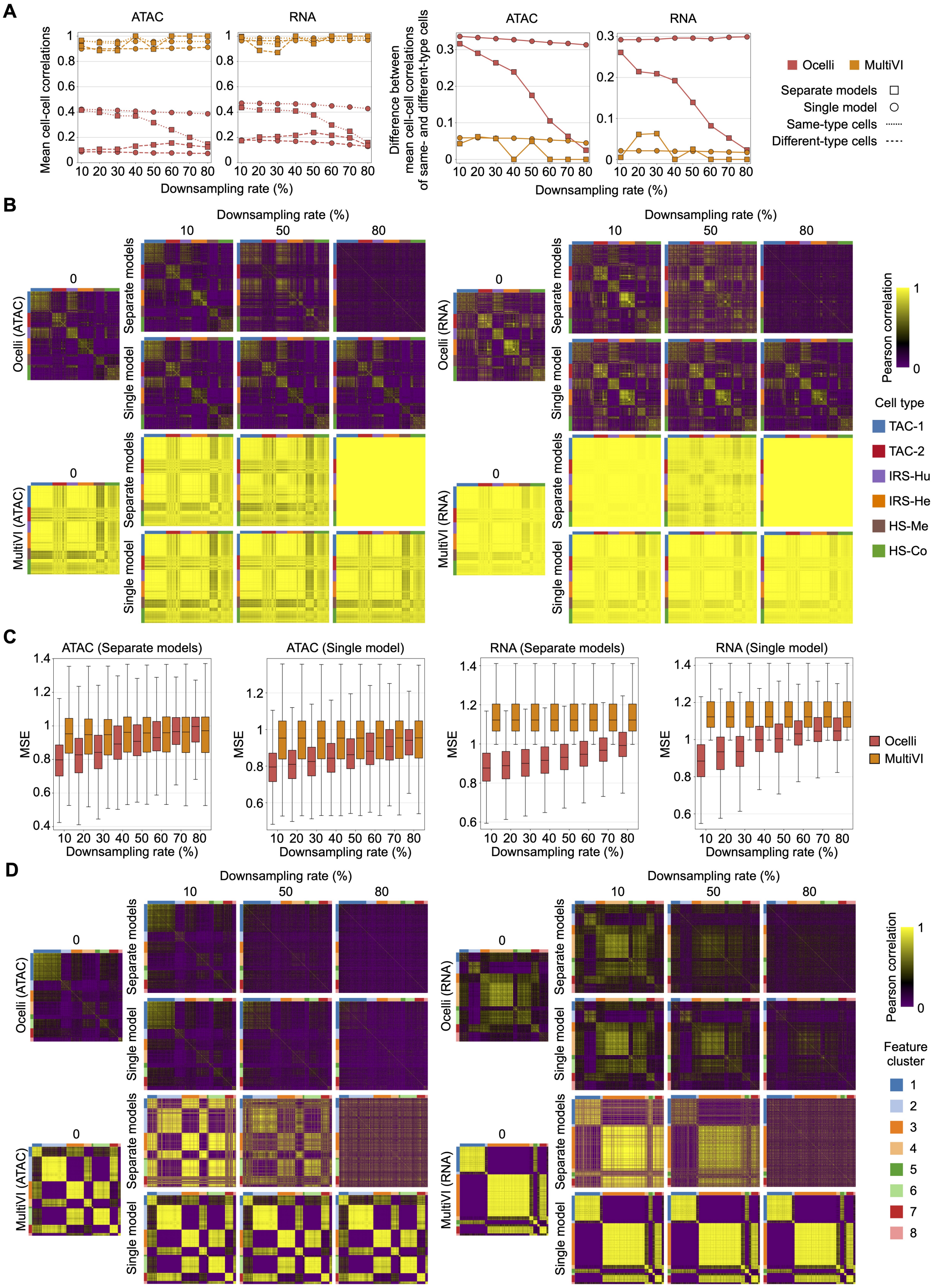

Figure S2. Additional results from multimodal imputation analysis (related to Figure 3). Analysis of cell-cell correlations in hair follicle SHARE-seq dataset imputed with Ocelli and MultiVI [16]. In *separate models* experiments, a separate model was trained for each downsampling rate. In *single model* experiments, a single model was trained on complete data without downsampling and then evaluated on imputed downsampled data. **(A)** Plots show mean cell-cell correlations for the same-type and different-type cells (left) and the difference between them (right). A higher difference indicates that the imputed data better preserves the relationships among cell types. **(B)** Cell-cell correlation matrices of the imputed downsampled data computed with Ocelli and MuliVI. **(C)** Mean squared errors (MSE) computed across features between imputed data and non-zero raw data counts show the accuracy of the imputation methods. **(D)** Feature-feature correlation matrices of imputed downsampled data with Ocelli and MuliVI. Features were clustered hierarchically into 8 clusters based on the imputed raw data.

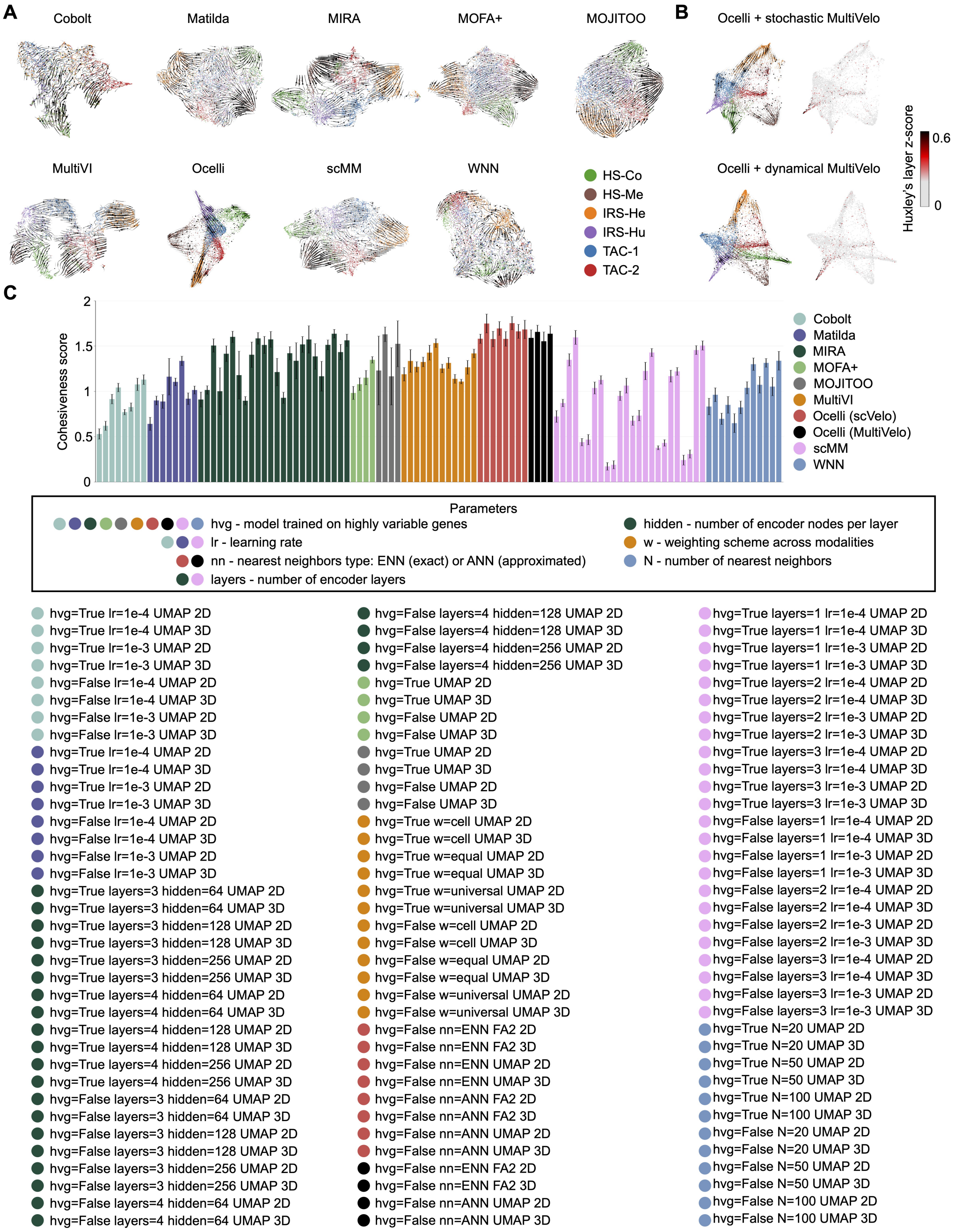

Figure S3. Additional results from benchmark analysis (related to Figure 4). **(A)** Scatter plots show scVelo's [7] RNA velocity stream projected onto 2D multimodal embeddings, generated in the analysis of hair follicle dataset by various methods. The embeddings presented scored the highest 2D vector field score for each method. **(B)** Ocelli embeddings boosted with MultiVelo [15] transition probabilities. Plots show velocity streams and z-scores of Huxley's layer gene signature [9]. **(C)** Cohesiveness scores of examined embeddings, ordered by method hyperparameters.

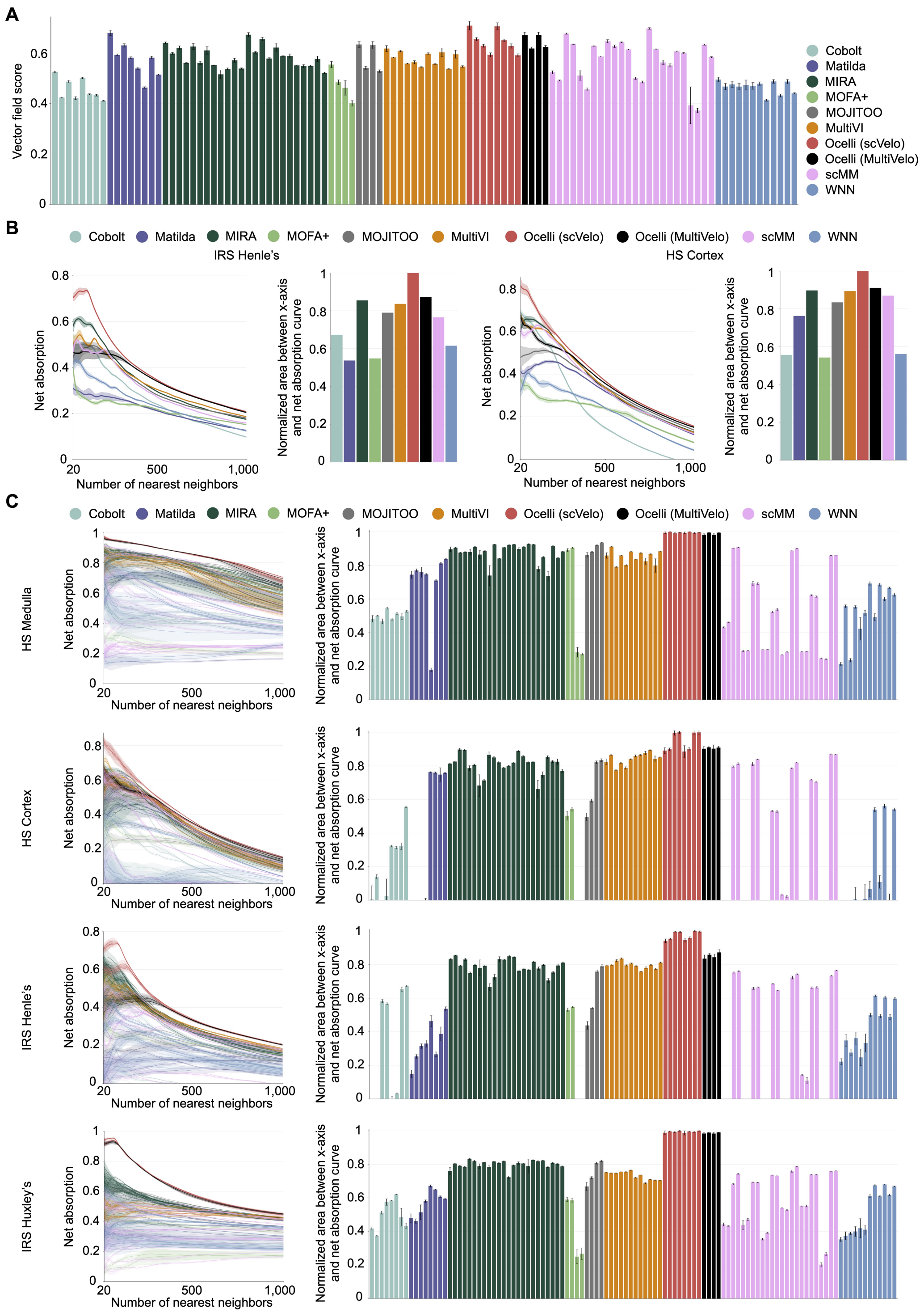

Figure S4. Additional results from benchmark analysis (related to Figure 4). **(A)** Vector field scores of examined embeddings, ordered by method hyperparameters (see Figure S3C). **(B)** Net absorption scores for inner root sheath Henle's layer (left) and hair shaft cortex (right) terminal states. Plots show best-performing models over hyperparameter search for each method. **(C)** Net absorption scores of examined embeddings, ordered by method hyperparameters (see Figure S3C).

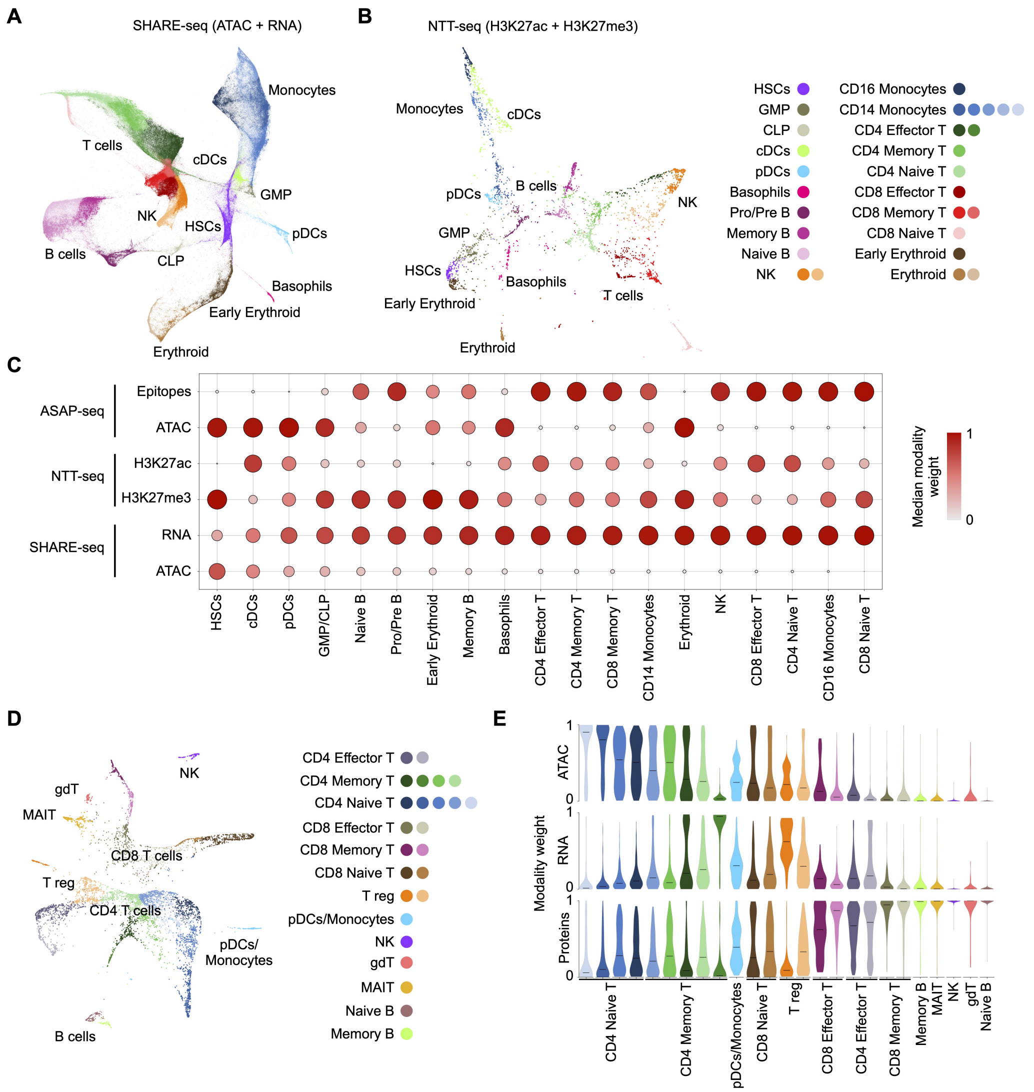

Figure S5. Supplementary analysis of native hematopoietic differentiation and peripheral blood mononuclear single-cell datasets. **(A-B)** Annotated visualizations of bone marrow mononuclear cells (BMMCs) datasets generated with Ocelli. **(A)** SHARE-seq [13] dataset contains 842,440 cells with profiled chromatin accessibility and transcriptome modalities. **(B)** NTT-seq [12] dataset contains 5,236 cells with H3K27ac and H3K27me3 modalities. **(C)** Multimodal weight distributions inferred from ASAP-seq (see Figure 5), NTT-seq and SHARE-seq BMMCs datasets. **(D)** Annotated visualization of single peripheral blood mononuclear cells (PBMCs) profiled with DOGMA-seq [11] method. The dataset has 7,516 cells with chromatin accessibility, transcriptome, and protein epitope modalities. **(E)** PBMCs DOGMA-seq multimodal weight distributions generated with Ocelli.
